# Retrospective Analysis of Factors Affecting Chronic Postoperative Pain After Thoracotomy: Single Center Experience

**DOI:** 10.4274/TJAR.2022.221059

**Published:** 2023-06-16

**Authors:** Nurlan Israfilov, Çiğdem Yıldırım Güçlü, Süheyla Karadağ Erkoç, Güngör Enver Özgencil

**Affiliations:** 1 Department of Anaesthesiology and Reanimation, Ankara University Faculty of Medicine, Ankara, Turkey

**Keywords:** Chronic pain, chronic pain risk factors, pain after thoracotomy, postoperative pain

## Abstract

**Objective:**

Despite various pain management methods, chronic pain is still a challenging issue after thoracotomy. This retrospective study was designed to determine the possible factors affecting the development of chronic pain following open thoracotomy.

**Methods:**

The study included patients who underwent elective open thoracotomy at Ankara University İbni Sina Hospital, between 01.01.2016 and 31.12.2020. The medical files and electronic records of the patients were scanned from the system. Patient history, analgesic methods, and surgical details were recorded. The need for and usage analgesic drugs after the surgery were also recorded.

**Results:**

A total of 229 patients who underwent thoracotomy were included in the study, and 83 (36.2%) patients had chronic pain. Duration of surgery, doses of remifentanil, fentanyl or NSAI drugs, duration or number of chest tubes (more than 4 days, or more than 2 tubes), diabetes, or PCEA usage were found as variables affecting pain. Logistic Regression, Multilayer Perceptron, Naive Bayes, AdaBoost, and Random Forest methods were used to evaluate the prediction performances. According to the model created with logistic regression, the rate of the correct classification was 90.8%. The duration of surgery, remifentanil administration, chest tube for more than 4 days, and diabetes were found to be risk factors for developing chronic pain. Fentanyl bolus, PCEA-bupivacaine, and NSAID bolus were determined as preventive factors.

**Conclusion:**

A careful analysis of risk factors should be performed for each patient to prevent chronic pain after thoracotomy, and preemptive effective analgesia methods should be performed.

**Main Points**

• Chronic pain after thoracotomy is not a rare complication

• Effective management of acute pain plays an important role in chronification of the pain

• Surgical duration plays an important role

• Intraoperative and postoperative continuous methods should be considered

## Introduction

Chronic pain lasts longer than the course of the disease and may persist for 3 to 6 months after recovery. It can be nociceptive, neuropathic, or mixed.^1^ Chronic pain following thoracotomy is not rare. The tissue around the surgical incision in thoracotomy is formed by musculoskeletal and nerve damage. It develops in two different periods: acute and chronic. If acute and severe pain that develops immediately after surgery is not adequately controlled or treated, the risk of developing chronic pain is high.^2^

Direct activation of nociceptors after surgery causes pain in and around the surgical site due to inflammation and nerve damage. Pain is associated with touch, movement, respiratory movements, or coughing. Additionally, it is thought that neuropathic components due to nerve damage develop after surgery, and thus pain may develop even in the absence of inflammation or peripheral nociception. Symptoms related to nerve damage are seen. Chronic pain that develops after surgery limits the daily activities of patients and adversely affects their quality of life.^3^

This retrospective study was designed to determine the factors influencing the development of chronic pain in patients undergoing thoracotomy at our center.

## Methods

This retrospective single-center study was conducted in Ankara University after ethical approval was obtained from the Human Research Ethics Committee of the Ankara University (Number: İ6-481-21). The clinical trial number for the study is NCT05501977. The study, initiated after the approval of the ethics committee dated 18.08.2021, was completed on 25.07.2022.

Patients with ASA I-III who were over 18 years of age and planned and scheduled for elective thoracotomy between 01.01.2016 and 31.12.2020 at Ankara University İbni Sina Hospital were included in the study. Patients with ASA IV and V, emergency or trauma thoracotomy, malignancy infiltrating the chest wall, more than one thoracotomy performed, cardiovascular, cerebrovascular, or musculoskeletal system affected at a level that would affect their daily activities after surgery, and patients with insufficient data for various reasons were excluded.

All patients’ medical files and electronic records were obtained from the hospital medical record system. Patients who presented to the pain clinic and reported pain at 3 and 6 months in the thoracic surgery outpatient clinic controls were classified as chronic pain group. Patients without pain at 3 and 6 months were classified as the non-chronic pain group. Age, sex, weight, ASA score, smoking, comorbidities, intraoperative and postoperative analgesia method, duration of acute pain, type of surgery, duration of surgery, duration of anaesthesia, rib resection, number and duration of chest tubes, time to apply to the pain clinic, and the analgesic method applied were recorded.

### Statistical Analysis

SPSS 11.5 was used in the analysis of retrospectively collected data. The mean ± standard deviation for quantitative variables and the number of patients (percentage) for qualitative variables were used as descriptors. To determine whether there is a difference between the categories of the qualitative variable with two categories in terms of quantitative variables, Student’s *t*-test was used if normal distribution assumptions were met, and the Mann-Whitney U test was used if assumptions were not met. Chi-square and Fisher's exact tests were used to examine the relationship between two qualitative variables. Risk factors affecting pain were examined using univariate logistic regression analysis. The statistical significance level was taken as 0.05. Logistic Regression, Multilayer Perceptron, Naive Bayes, AdaBoost, and Random Forest techniques were used for the machine learning classification. The dataset was tested using 10-fold cross validation. Accuracy, F-measure, Matthews correlation coefficient (MCC), ROC curve, and precision recall curve (PRC) were used as performance criteria. All analysis were made using the R programming language, and the RWeka and e1071 packages in the language were used with this program.

## Results

Between 01.01.2016 and 31.12.2020, a total of 326 patients who underwent thoracotomy at İbn-i Sina Hospital were initially examined. Of the patients examined, thirty-one patients were excluded because of missing data, nineteen patients due to ASA IV and V, seventeen patients due to death, twelve patients under the age of 18, and 8 patients due to emergency trauma surgery. A total of 229 patients who underwent thoracotomy was included in the study.

While 146 (63.8%) patients had no complaints of chronic pain, 83 (36.2%) patients had chronic pain. When examining whether there was a difference between chronic pain categories in terms of demographic and clinical data, a significant difference was found in terms of hypertension and diabetes; 45.8% of patients with hypertension and 59.6% of patients with diabetes had chronic pain (Table 1).

We examined whether there was a difference in the development of chronic pain in terms of the variables of the intraoperative and postoperative medical analgesia methods. While 64.6% of patients who were administered remifentanil had chronic pain, this rate was 21.3% of patients who received fentanyl. Chronic pain was present in 23.6% of patients who were administered lidocaine infusion. A total of 44.2% of the patients who underwent IV fentanyl-PCA had chronic pain, and it was determined that 29.5% of the patients who were treated with non-steroidal anti-inflammatory drugs (NSAIDs) had chronic pain. The patients (9.1%) who received epidural bupivacaine-PCA had chronic pain, while this rate was 44.8% in patients who did not receive epidural bupivacaine-PCA (Table 2).

The mean duration of surgery was significantly higher in patients with chronic pain. Rib resection is an important factor for chronic pain, 85.7% of patients with rib resection have chronic pain. Number of chest tube and chest tube duration time were found to be significantly higher in patients with chronic pain. The mean duration of acute pain was 4.29±1.60 days in patients with chronic pain and 3.71±1.34 days in patients without chronic pain (Table 3).

The risk factors affecting chronic pain were examined by logistic regression analysis, and hypertension and diabetes were found to be significant risk factors. The presence of hypertension increases the risk of chronic pain by 1.895 times, and the presence of diabetes increases the risk of chronic pain by 3.403 times.

The risk of chronic pain increases by 6,717 times in patients not using fentanyl. The risk of chronic pain increases by 2.175 times in patients who do not use lidocaine infusion. While the risk of chronic pain increases by 8.125 times in patients not using epidural bupivacaine-PCA. The use of NSAIDs also effects chronic pain. Increasing the duration of acute pain by one day increases the risk of chronic pain by 1,312 times, while increasing the duration of surgery by one minute increases the risk of chronic pain by 1,058 times. The presence of rib resection increases the risk of chronic pain by 11,299 times. Increasing the number of chest tubes by one increases the risk of pain by 4,742 times, and an increase in the duration of the chest tube by one day increases the risk of chronic pain by 1.747 times (Table 4).

**Figure 1:**
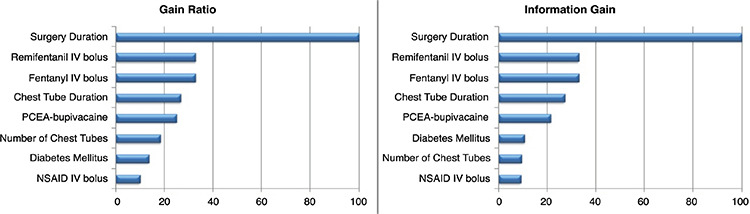
Variable Importances based on Gain Ratio and Information Gain Methods.

As to decide the importance of the variable and the effect of the variables on pain were examined more tests performed (the gain ratio and information gain variable significance tests) (Figure 1). When the significance of the variable was evaluated according to the results of the tests and clinical significance, the model consisted of surgery duration, remifentanil IV bolus, fentanyl IV bolus, chest tube duration, PCA-bupivacaine, number of chest tubes, diabetes mellitus, NSAID IV bolus. As a result, 9 variables (8 independent, 1 dependent variable) were included in the study and machine learning analyzes were performed using these variables.

**Figure 2:**
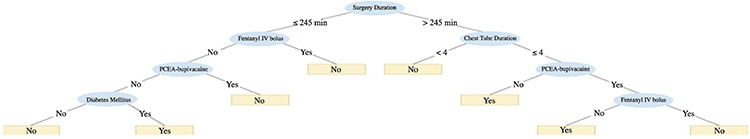
A Tree Diagram of the Random Forest Method.

Logistic regression, multilayer perceptron, naive Bayes, AdaBoost and random forest methods were used to evaluate the prediction performances, and the results of these methods are given in Table 1. Logistic Regression gave the best results according to the correct classification rate, F-criterion and MCC, which are among the most frequently used performance measures in the literature. This method was followed by AdaBoost, Naive Bayes, Multilayer Perceptron and Random Forest methods, respectively. The diagram of one of the trees created in Random Forest is given in Figure 2.

According to the model created with logistic regression, the rate of correct classification was 90.8%. With this model, when we made a general estimation of no/has pain for the patient, the accuracy rate of this estimation was 90.8%. In other words, with this model, the prediction result of about 90 out of 100 people was correct. In addition, the correct classification rate of the patients who said they had no pain in this model was 93.8%, and the rate of accurate classification of the patients who said they had pain was 85.5% (Table 5).

## Discussion

The incidence of CPAT is high. In different studies, the prevalence of chronic pain developing after thoracotomy varies between 25% and 68%.^4,5^ In our study, the incidence of CPAT was 36.2%, which was consistent with the literature.

Advanced age is considered a risk factor for many diseases, but many studies have shown that patients who develop CPAT are at least 10 years younger than patients who do not develop pain and that the risk of developing pain is higher in patients under 60 years of age.^6^ The higher risk in younger patients is thought to be due to stronger inflammatory and immune responses. In our study, age was not found to be one of the risk factors for CPAT because the majority of patients included in the study were elderly.

Different experimental studies have shown that there is a relationship between pain and hypertension. In studies, different mechanisms come into play to prevent and regulate blood pressure in hypertensive patients who develop pain. One of these mechanisms is the excessive release of endogenous opioids to control pain. Because endogenous opioid secretion is higher in hypertensive patients than in healthy individuals, tolerance develops after a certain period, and it is thought that an increase in pain sensitivity can be observed.^7^ In our study, CPAT developed in 45.8% of patients with hypertension, and hypertension increased the risk of pain development by 1.9 times.

Polyneuropathy causing severe pain is seen in patients with diabetes. Complex mechanisms involving inflammation, microvascular, and immune responses contribute to the development of neuropathic pain in diabetic patients. Neuropathic pain developing before surgery is thought to trigger the development of chronic pain in the postoperative period together with surgical stress.^8^ ASA I-IV patients over the age of eighteen who had undergone thoracotomy at Peking Union Medical College Hospital between 2009 and 2020 were examined. They found that the risk of developing chronic pain in patients with diabetes was significantly higher than that in patients without diabetes.^9^ In our study, the results were similar, and the risk of developing CPAT was found to be 3,403 times higher in patients with diabetes.

It is thought that the use of intraoperative remifentanil causes the development of hyperalgesia in the postoperative period and pain that develops in the acute period affects the development of chronic pain. In this study, the effect of intraoperative remifentanil and fentanyl use on chronic pain after cardiac surgery was investigated. A randomized controlled study by de Hoogd et al.,^10^ 126 patients were included, and the rate of chronic pain development at the third month after surgery was found to be significantly higher in the remifentanil group compared to the fentanyl group. In our study, chronic pain was observed in 21.3% of the patients who received fentanyl, whereas the rate was found to be 64.5% in patients who used remifentanil. Intraoperative use of remifentanil increased the risk of chronic pain by 6.7 times.

Lidocaine infusion, which is used in multimodal analgesia, prevents chronic pain that develops after surgery by different mechanisms. Its ability to prevent chronic pain is due to its sodium channel blockade in neurons, its ability to prevent inflammation, and its antihyperalgesic effects. In the study by Terkawi et al.,^11^ which included sixty-one patients who underwent mastectomy, the patients were divided into two groups: placebo was administered to one group and intraoperative lidocaine infusion was administered to the other group. Because of the study, chronic pain developed in 30% of patients who received placebo at the 6^th^ month after surgery, but this rate was found to be significantly lower in patients who received lidocaine infusion (12%).^11^ Our results were similar; chronic pain developed in 23.6% of patients who received lidocaine infusion, but chronic pain developed in 40.3% of patients who did not receive lidocaine infusion.

Different treatment methods are used for pain control after thoracotomy. Among the multimodal treatment methods, intravenous and epidural patient-controlled analgesia methods are frequently and widely used. Li et al.^12^ compared IV-PCA and epidural-PCA methods. Ninety-six patients who had undergone thoracotomy were included in the study, and the patients were divided into two groups: intravenous and continuous epidural analgesia. Because of the study, the rate of development of chronic pain in the third month was 55.2% in patients who received IV analgesia and 28.6% in patients who received continuous epidural analgesia.^12^ In our study, it was determined that chronic pain developed in 44.2% of patients who received IV fentanyl-PCA chronic pain developed in 9.1% of patients who received epidural bupivacaine-PCA.

There are many studies on the relationship between acute postoperative pain and chronic postoperative pain. This relationship was shown in the study of Kalso et al.^13^ in 1992 for the first time. The development of acute postoperative pain is a risk factor for postoperative chronic pain.^13^ Wang et al.^9^ investigated the risk factors affecting the development of chronic pain after thoracotomy. A total of 466 patients who underwent thoracotomy between 2009 and 2010 were included in the study, and it was found that each additional day of acute pain duration increased the risk of developing chronic pain by 1.2 times.^9^ Similar results were obtained in our study. It was determined that increasing the duration of acute pain for one day increased the risk of chronic pain by 1,312 times, and more research is needed on this subject.

Neurological damage during surgery is thought to be the source of postoperative chronic pain. Rib resection and suturing techniques increase the risk of nerve damage.^14^ In the study of Maguire et al.,^15^ it was determined that patients who did not undergo rib resection were exposed to more intercostal nerve damage during rib resection. Although the effect of rib resection on the development of postoperative chronic pain is controversial, there are studies with different results. In a study that retrospectively investigated the risk factors affecting the development of chronic pain after thoracotomy and included 208 patients, it was found that the risk of developing chronic pain increased by 9.8 times in patients who underwent rib resection.^16^ In our study, rib resection increased the risk of chronic pain after surgery by 11.8 times.

The number of chest tubes placed after surgery is believed to influence the development of chronic pain. As the number of tissue damage increases, the risk of developing chronic pain after surgery also increases. In the study of Miyazaki et al.,^17^ it was determined that intercostal nerve damage developed during chest tube insertion, with the risk increasing as the number of procedures increases. Mongardon et al.^6^ included sixty-eight patients who underwent posterolateral thoracotomy between 2007 and 2008, in which 48% of the patients developed chronic pain. It was determined that the risk of developing chronic pain increased as the number of chest tubes increased.^6^ The results of this study were similar, the risk of developing CPAT increased by 4.7 times in patients with two chest tubes compared with patients with one chest tube.

The risk of developing CPAT increases as the duration of the chest tube increases because of continuous exposure to tissue damage. In a retrospective study by Kar et al.^16^ that included 208 patients in which the risk factors affecting the development of chronic pain after thoracotomy were investigated, it was found that the risk of developing CPAT increased as the duration of chest tube thrusting increased. In another study, risk factors affecting the development of chronic pain after thoracotomy was investigated. A total of 466 patients who had undergone thoracotomy between 2009 and 2010 were included in the study, and it was found that the risk of developing CPAT increased by 1.3 times as the duration of chest tube stay increased.^14^ Similar results were obtained in our study, and it was determined that the risk of chronic pain increased by 1.747 times with each additional day of chest tube insertion.

After more detailed analysis of the statistics for related factors, the duration of surgery, remifentanil administration, chest tube existence for more than 4 days, and the presence of diabetes were found to be more related risk factors for the development of chronic pain than the others that were evaluated. The administration of a fentanyl bolus, PCEA-bupivacaine, and a NSAID bolus were determined as preventive factors.

The duration of surgery was found to be the most related factor to the development of chronic pain. Yoon et al.^18^ evaluated 3200 patients after thoracic surgery, which covers a 10-year period. They concluded that the duration of surgery is a factor that relates to chronic pain, which is consistent with our results. The continuous and prolonged surgical impulses may explain these findings.^18^

### Study Limitation

A limitation of the study is that as this is a retrospective study, it is not possible for us to form groups and compare methods. This study may guide us to establish a new prospective comparative study to determine an effective approach in the preventing of chronic pain in thoracotomy patients.

## Conclusion

The development of chronic pain after thoracotomy is an important health problem influenced by multiple factors rather than a single cause. Because of our research, hypertension, diabetes, intraoperative intravenous remifentanil infusion, rib resection, prolongation of acute pain duration, number of chest tubes and prolonged chest tube duration were determined to be risk factors for the development of chronic pain after thoracotomy. Intraoperative fentanyl and lidocaine infusion, postoperative epidural bupivacaine-PCA, and NSAID uses have been found to significantly reduce the development of chronic pain after thoracotomy. Duration of surgery, remifentanil administration, chest tube existence for more than 4 days, and diabetes were found to be more related to chronic pain after thoracotomy. By identifying these factors, we applied the necessary treatments to prevent the development of chronic pain and improve patient outcomes.

## Figures and Tables

**Table 1:**
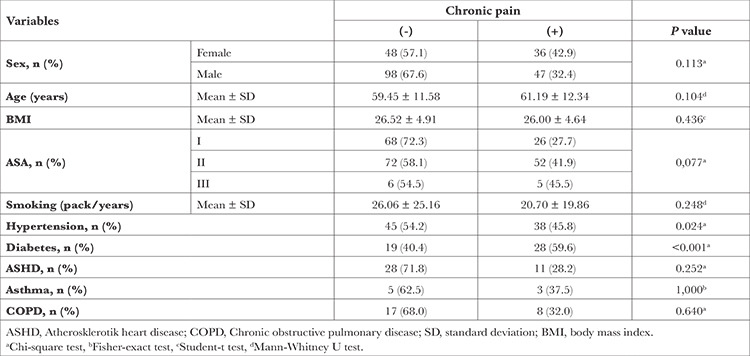
Comparisons of Demographic and Clinical Characteristics for Chronic Pain After Thoracotomy

**Table 2:**
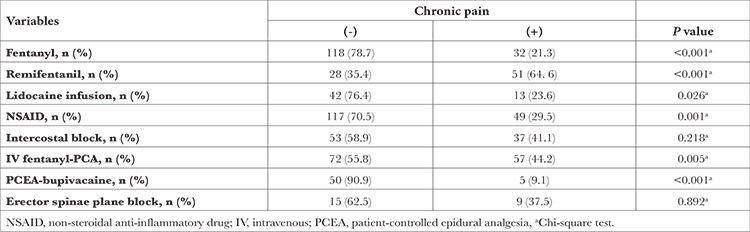
Comparison of Variables of Intraoperative and Postoperative Analgesic Methods for Chronic Pain After Thoracotomy

**Table 3:**
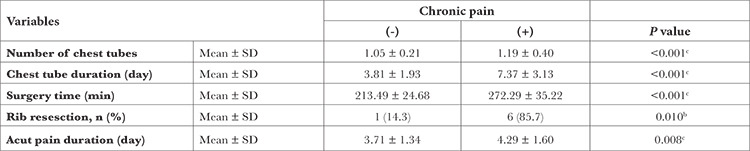
Conditions of Variables for Intraoperative and Postoperative Surgical Procedures for Chronic Pain After Thoracotomy

**Table 4:**
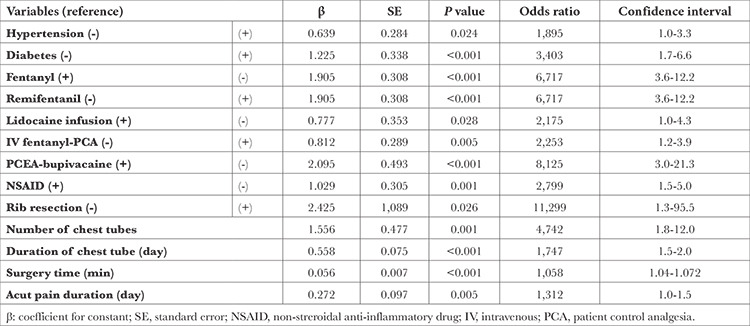
Logistic Regression Analysis for Factors Affecting Chronic Pain After Thoracotomy

**Table 5:**
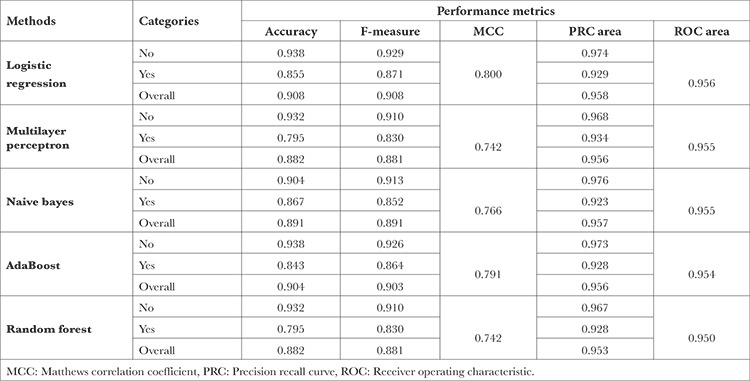
Performance Metrics of Machine Learning Models
